# Prevalence, bacterial load, and genomic characterization of *Vibrio parahaemolyticus* in freshwater-farmed *Litopenaeus vannamei* from China

**DOI:** 10.3389/fmicb.2025.1713237

**Published:** 2025-12-10

**Authors:** Feifei Shan, Weiwei Li, Zunhua Chu, Peibin Hou, Na Sun, Ronghua Li, Xuejie Liu, Weiwei Chen, Ling Zhong, Shaofei Yan, Shenghui Cui, Yunchang Guo

**Affiliations:** 1 Institute for Nutrition and Health, Chinese Center for Disease Control and Prevention, Beijing, China; 2 Key Laboratory of Food Safety Risk Assessment, Food Safety Research Unit (2019RU014) of Chinese Academy of Medical Science, China National Center for Food Safety Risk Assessment, Beijing, China; 3 Shandong Center for Disease Control and Prevention, Jinan, China; 4 Jining Center for Disease Control and Prevention, Jining, China; 5 Fujian Center for Disease Control and Prevention, Fuzhou, China; 6 Zhangzhou Center for Disease Control and Prevention, Zhangzhou, China; 7 National Institutes for Food and Drug Control, Beijing, China

**Keywords:** *Vibrio parahaemolyticus*, *Litopenaeus vannamei*, prevalence, bacterial load, genomic analysis

## Abstract

Shrimp was the leading food vehicle for *Vibrio parahaemolyticus* outbreaks in China, yet studies on *V. parahaemolyticus* in globally predominant farmed shrimp (*Litopenaeus vannamei*) - particularly in freshwater culture remain limited. This study aimed to investigate and evaluate the contamination of *V. parahaemolyticus* in freshwater-farmed marine shrimp (*L. vannamei*) in China. The prevalence and bacterial load of *V. parahaemolyticus* in shrimps were assessed by China China National Standard GB 4789.7-2013 and isolates were obtained for genomic analysis. The prevalence of *V. parahaemolyticus* in shrimp samples was 39.1% and the bacteria load ranged from 3.6 MPN/g to 24,000 MPN/g. Analysis on isolates demonstrated that 75.8% (94/124) were assigned to 28 sequence types (STs), whereas 24.2% STs were unknown. All isolates harbored 24 antibiotic resistance genes (ARGs), of which *tet(34)*, *tet(35)*, and *bla*_CARB_ were harbored in all isolates. There was a significant correlation between *bla*_CARB_ types and STs, with isolates sharing identical STs carried the same blaCARB variant. The *trh*+ isolates (9.7%, 12/124) was simultaneously coexisted with *hlyB* and *hlyC*. All four ST79 strains harbored *trh*/*hlyB*/*hlyC* and *bla*_CARB_46_. Our findings elucidated the contamination of *V. parahaemolyticus* in freshwater-farmed *L. vannamei*, with heavy bacteria load in Fujian province. The emergence of specific ST co-harboring critical virulence genes and ARGs indicating the necessity for targeted surveillance and specified control in aquaculture systems.

## 1 Introduction

*Vibrio parahaemolyticus* can cause acute gastroenteritis in humans through contamination of raw or undercooked aquatic products (Ceccarelli et al., 2013; Ghenem et al., 2017; Su and Liu, 2007). The primary implicated food for *V. parahaemolyticus* outbreaks in America and Europe is shellfish (Cabello et al., 2007; Centers for Disease Control and Prevention, 1998, 1999; Fearnley et al., 2024; Lozano-León et al., 2003; Raszl et al., 2016; Taylor et al., 2018). Meanwhile, according to China national foodborne disease surveillance, the primary food is shrimp. According to FAO (2024) and China Fisheries Statistical Yearbook (2023), *Litopenaeus vannamei* ranked top in shrimp aquaculture production globally, including China. Although *L. vannamei* is usually produced via marine aquaculture, its farming differs in China. More than 36% of total *L. vannamei* production was transferred to freshwater farming in China. Although studies on *V. parahaemolyticus* contamination in marine-farmed *L. vannamei* are not rare, studies investigating *V. parahaemolyticus* contamination in freshwater-farmed *L. vannamei* i are scarce, studies on their genetic traits are even rarer.

Although *V. parahaemolyticus* has a wide array of virulence genes, including hemolysins and type III secretion systems (T3SS), the profile of virulence genes varies significantly among different source isolates. Notably, the thermostable direct hemolysin (TDH) and TDH-related hemolysin (TRH), which are encoded by *tdh* and *trh* genes, respectively, have clinical significance and serve as key virulence markers (Nichuchi and Kaper, 1995; Raghunath, 2015). In addition, *V. parahaemolyticus* develops resistance to multiple antibiotics owing to the misuse of antibiotics in aquaculture production (Elmahdi et al., 2016; Hossain et al., 2022). Studies from regions such as Bulgaria, South Korea, Mexico, China, Vietnam, and Malaysia have demonstrated that *V. parahaemolyticus* isolates from aquatic products are resistant to multiple antibiotics, including ampicillin, ciprofloxacin, ceftazidime, cefotaxime, and tetracycline. Among these, the ampicillin-resostant strains accounted for the highest proportion (Flores-Villaseñor et al., 2024; Jeong et al., 2020; Li et al., 2020; Stratev et al., 2023; Tan et al., 2020; Vu et al., 2022).

Therefore, this study aimed to investigate the contamination status and assess the virulence and antimicrobial resistance potential of *V. parahaemolyticus* in freshwater-farmed marine shrimp (*L. vannamei*) in China.

## 2 Materials and methods

### 2.1 Sample collection

Samples were collected from nine sampling sites across freshwater *L. vannamei* farms in the Shandong and Fujian provinces of China, including *L. vannamei* (184), freshwater (42), feed (18), and aquaculture worker’s feces (6). From each sampling pool, approximately 100 g of *L. vannamei* shrimp samples and 450 mL of culture water samples were collected. Water temperature and salinity were simultaneously measured. Detailed information are presented in Supplementary material.

### 2.2 Detection and isolation

*V. parahaemolyticus* was qualitatively and quantitatively detected in samples following the Chinese national standard GB 4789.7 - 2013 “Microbiological Examination of Food - Detection of *Vibrio parahaemolyticus*.” All samples underwent primary enrichment in 3% sodium chloride alkaline peptone water with incubation at 37 °C for 12 h. Subsequent *V. parahaemolyticus* isolation was achieved by streaking onto selective thiosulfate-citrate-bile salts-sucrose (TCBS) agar plates and incubated at 37 °C for 24 h. Typical colonies were cultured onto 3% sodium chloride tryptic soy agar plates at 37° C for 24 h. Presumptive *V. parahaemolyticus* isolates were confirmed through Vitek 2 Compact. Afterwards, the confirmed *V. parahaemolyticus* strains were then taken for whole genome sequencing.

### 2.3 Whole genome sequencing

Genomic DNA was extracted using a Bacterial Genomic DNA Extraction Kit (Beijing Tiangen Biotech Co., Ltd.). The extracted DNA was sent to Novogene for whole-genome sequencing on an Illumina NovaSeq 6000 platform using the Illumina HiSeq protocol. Genome assembly used SPAdes v3.15.4.^1^ Quality control of the assembled sequences was performed using CheckM.^2^ Duplicate strains were screened by integrating Average Nucleotide Identity (ANI) analysis^3^ and QUAST.^4^ The reference strain used for comparison was *V. parahaemolyticus* ATCC17802.

### 2.4 Bacterial species identification and multi-locus sequence typing

Bacterial species were identified using KmerFinder 3.2.^5^ Multi-locus sequence typing (MLST) of the strains was conducted based on seven housekeeping genes (dnaE, recA, dtdS, gyrB, pntA, tnaA and pryC) using PubMLST.^6^ A minimum spanning tree was constructed using GrapeTree’s MSTree algorithm^7^ based on multi-locus sequence typing (MLST).

### 2.5 Screening of ARGs, virulence genes, and plasmids

ARGs, virulence genes and plasmids were identified using the ResFinder,^8^ Virulence Factor Database (VFDB^9^) and PlasmidFinder.^10^ For all analyses, minimum thresholds of ≥80% coverage and ≥80% identity were applied.

### 2.6 Phylogenetic analysis

Genome annotation was performed using Prokka v1.14.6. Core genome analysis was perforemed using Roary v3.13.0 and Mafft v7.525. The core SNPs were identified using Snippy v4.6.0.^11^ Gubbins v2.4.1 was used to filter SNP recombination.^12^ A maximum-likelihood phylogenetic tree was generated using FastTree v2.1.10 with parameters “nt -gtr.” Phylogenetic tree of the genomes was visualized using FigTree v1.4.4.^13^

## 3 Results

### 3.1 Characteristics of sampling sites

The sampling months and environmental parameters (e.g., water temperature and salinity) of aquaculture farms in Shandong and Fujian are shown in Table 1. The average water temperature and salinity in aquaculture farms in Shandong and Fujian provinces were 32.8 °C, 0.53‰ and 24.2 °C, 3.65‰, respectively.

**Table 1:** Basic information on sampling of *Litopenaeus vannamei* farms.

Province	Sampling time	Temperature (°C)	Salinity (‰)
Shandong	June 2024; August 2024	24.7 ~ 36.5	0.4 ~ 0.8
Fujian	December 2023; March 2024	19.0 ~ 29.0	2.0 ~ 8.0

### 3.2 Prevalence and bacterial load of *Litopenaeus vannamei*

**Figure 1:**
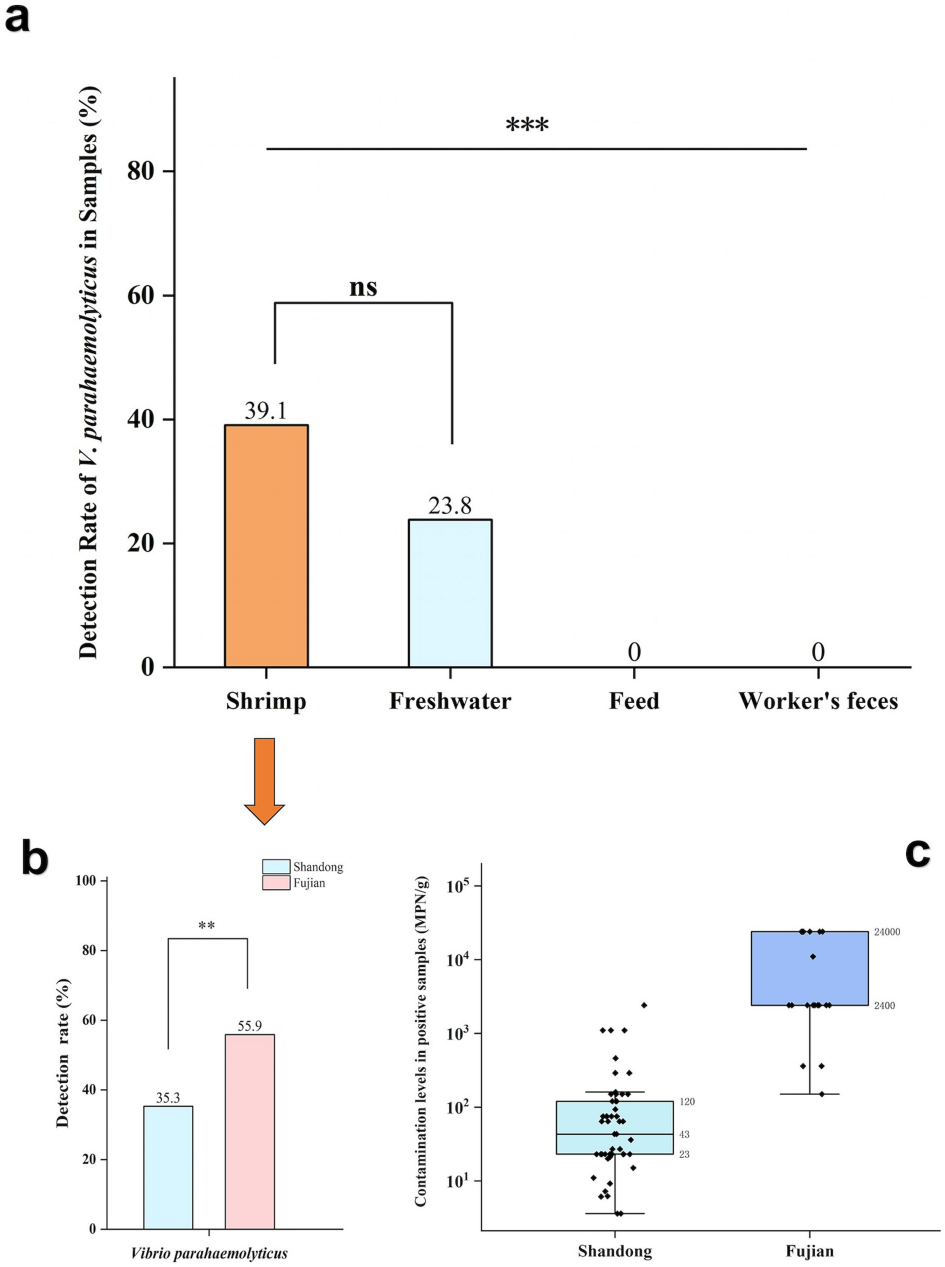
Detection and bacterial load of *V. parahaemolyticus* in samples. **(a)** Detection of *V. parahaemolyticus* in different type samples types. **(b)** Detection of *V. parahaemolyticus* in samples of shrimp from different provinces. **(c)** Contamination levels of *V. parahaemolyticus* in positive samples of shrimp from different provinces. ‘**’ means *P* < 0.01, ‘***’ means *P* < 0.001, ‘ns’ means *P* > 0.05.

A total of 250 samples were collected, comprising 184 shrimp samples, 42 aquaculture freshwater samples, six worker’s feces samples, and 18 feed samples. The overall detection rate of *V. parahaemolyticus* was 32.8% (82/250), with detection rates of 39.1% (72/184) in shrimp and 23.8% (10/42) in freshwater samples. *V. parahaemolyticus* was not detected in workers’ feaces or feed samples. There was a statistically significant difference in *V. parahaemolyticus* detection rates among the sample types (*χ*^2^ = 23.958, *p* < 0.05) (Figure 1a).

This study was conducted in Shandong and Fujian provinces. The overall detection rate of *V. parahaemolyticus* was 26.7% (54/202) in Shandong and 58.3% (28/48) in Fujian, and the difference was statistically significant (*χ*^2^ = 17.571, *p* < 0.05). For shrimp samples, the detection rates of *V. parahaemolyticus* were 35.3% (53/150) in Shandong and 55.9% (19/34) in Fujian, with a statistically significant difference (*χ*^2^ = 4.914, *p* < 0.05). The median (P_25_, P_75_) pollution levels of *V. parahaemolyticus*-positive shrimp samples in Shandong and Fujian were 43 (23, 120) MPN/g and 2,400 (2,400, 24,000) MPN/g respectively, with a statistically significant difference in pollution levels (*Z* = − 6.145, *p* < 0.05) (Figures 1b,c).

### 3.3 MLST and the minimum spanning tree

**Figure 2:**
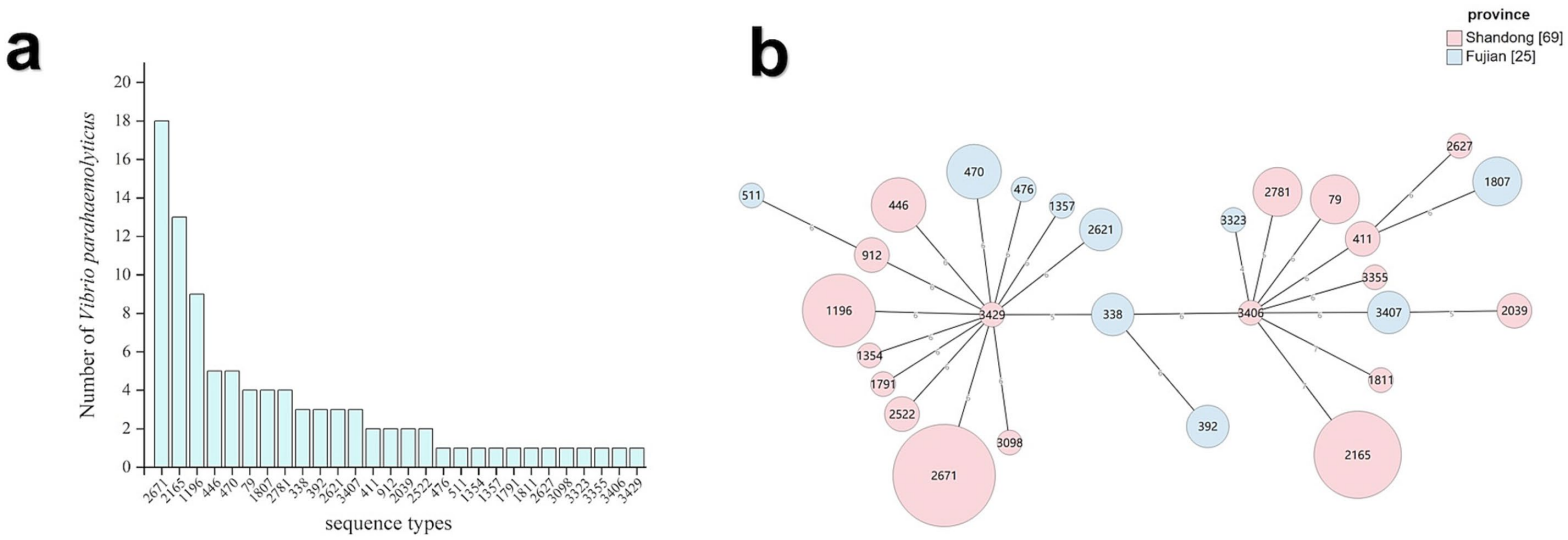
MLST and minimum spanning tree of 94 *V. parahaemolyticus* isolates. **(a)** Distribution of MLST in 94 isolates. **(b)** Minimum spanning tree of 94 isolates.

Of the 124 *V. parahaemolyticus* strains, 94 were assigned to 28 STs, with 16 STs containing at least two isolates each, whereas 12 STs were represented by single isolates only. Predominant STs were ST2671 (19.6%, 18/92), ST2165 (14.1%, 13/92), and ST1196 (9.8%, 9/92) (Figure 2a). The minimum spanning tree based on provincial origins and allelic profiles revealed two distinct clusters with clear genetic structures (Figure 2b). The distribution of sequence types in the shrimp and water isolates is shown in Supplementary material.

### 3.4 Distribution of ARGs and plasmids

**Figure 3:**
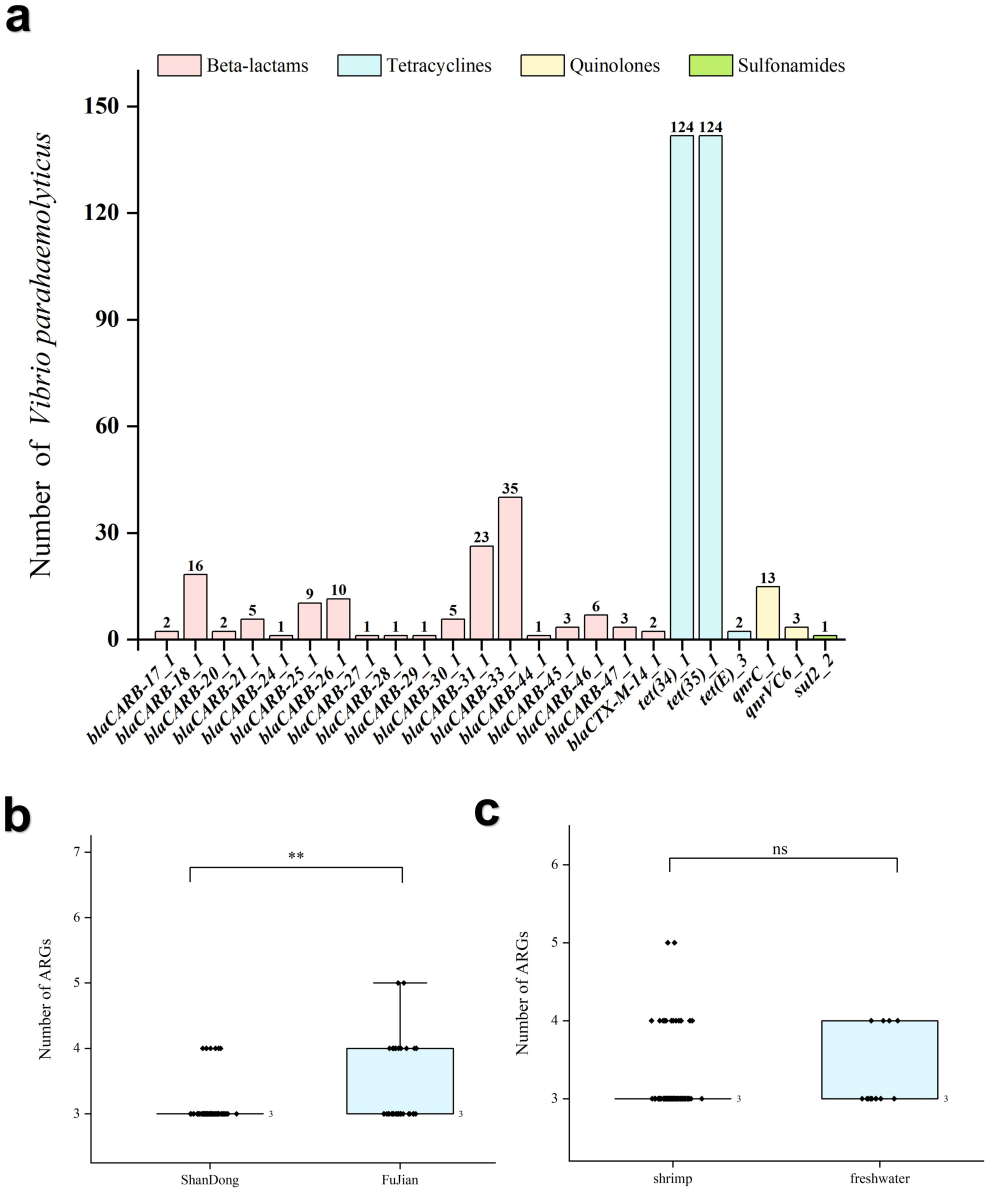
Distribution and statistical analysis of ARGs in 124 *V. parahaemolyticus* isolates. **(a)** Distribution of ARGs in 124 *V. parahaemolyticus* isolates. **(b)** Differences in the number of ARGs in isolates across different provinces. **(c)** Differences in isolates across number of ARGs in different sources. ‘**’ means *P* < 0.01, ‘***’ means *P* < 0.001, ‘ns’ means *P* > 0.05.

In this study, a total of 24 ARGs were identified in 124 *V. parahaemolyticus* strains and classified into four antimicrobial categories, namely β-lactam, tetracycline, quinolone, and sulphonamide (Figure 3a). Notably, *tet(34)*, *tet(35)*, and *bla*_CARB_, responsible for tetracycline and β-lactam resistance, were present in all the 124 isolates. Additionally, only a small proportion of the isolates carried resistance genes against quinolone and sulphonamide antibiotics, with detection rates of 12.9% (16/124) and 0.8% (1/124), respectively. A *lncC*-type plasmid was identified in one shrimp-derived isolate; however, no known antibiotic resistance genes were detected in this plasmid sequence.

The 124 strains were classified by provinces (77 from Shandong and 44 from Fujian) and sources (110 from shrimp and 14 from aquaculture water). The Mann–Whitney U test revealed a statistically significant difference in the number of ARGs between different provinces (*Z* = −2.769, *p* < 0.05). In contrast, there was no significant difference between shrimp and aquaculture water isolates (*Z* = −1.287, *p* = 0.198) (Figures 3b,c).

### 3.5 Distribution of virulence genes

**Figure 4:**
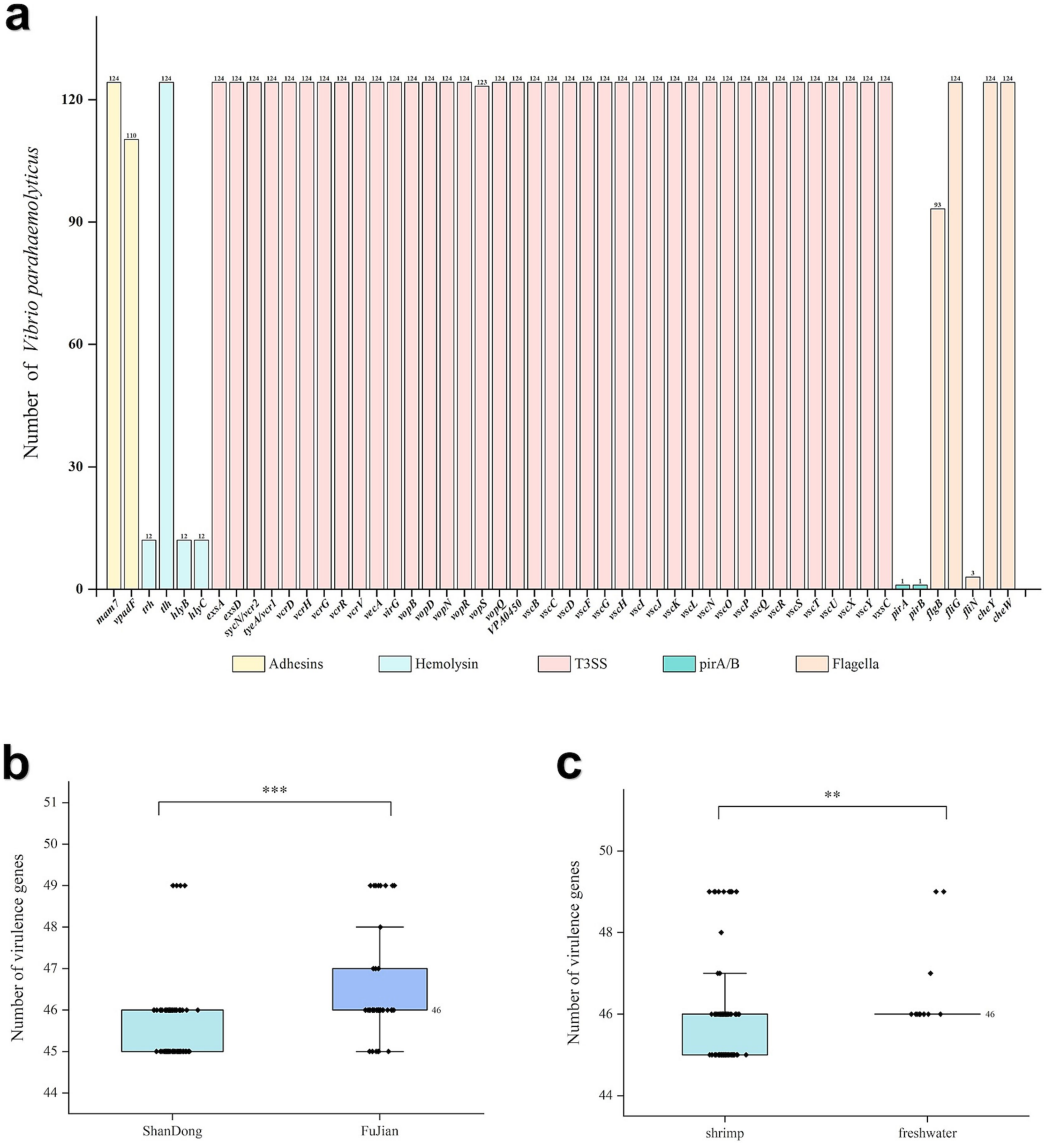
Distribution and statistical analysis of virulence genes in 124 *V. parahaemolyticus* isolates. **(a)** Distribution of virulence genes in 124 *V. parahaemolyticus* isolates. **(b)** Differences in the number of virulence genes in isolates across different provinces. **(c)** Differences in the number of virulence genes in isolates across different sources. ‘**’ means *P* < 0.01, ‘***’ means *P* < 0.001, ‘ns’ means *P* > 0.05.

Among the 124 *V. parahaemolyticus* strains analyzed, a total of 52 virulence genes encoding five pathogenic factors were identified. Among these, 50 genes related to hemolysin, Type III Secretion System (T3SS), adhesion factors, and flagella have the potential to pose risks to human health. Notably, the *pirA* and *pirB* genes, encoding the toxins PirA and PirB, respectively, are strongly linked to acute hepatopancreatic necrosis disease (AHPND) in shrimp. Four hemolysin-related genes were detected, with all the strains harboring the *tlh* gene and 9.7% (12/124) of the strains harboring the *trh*, *hlyB*, and *hlyC* genes. All the strains possessed T3SS1-related genes, with 99.2% (123/124) encoding four T3SS1 effector proteins (*vopR*, *vopS*, *vopQ*, and *VPA450*). Additionally, two adhesion genes (*mam7* and *vpadF*) and five flagella genes (*fliG*, *cheY*, *cheW*, *flgB*, *fliN*) were also identified (Figure 4a).

In this study, most of the strains (87.1%, 108/124) harbored 45 or 46 virulence genes. The median numbers of virulence genes carried by strains from Shandong and Fujian provinces was 45 and 46, respectively, and the difference in the number of virulence genes harbored by strains from the two provinces was statistically significant (*Z* = −4.774, *p* < 0.05). In addition, the differences in the number of virulence genes harbored by strains from different sources remained significant (*Z* = −2.824, *p* < 0.05) (Figures 4b,c).

### 3.6 Phylogenetic analysis

**Figure 5:**
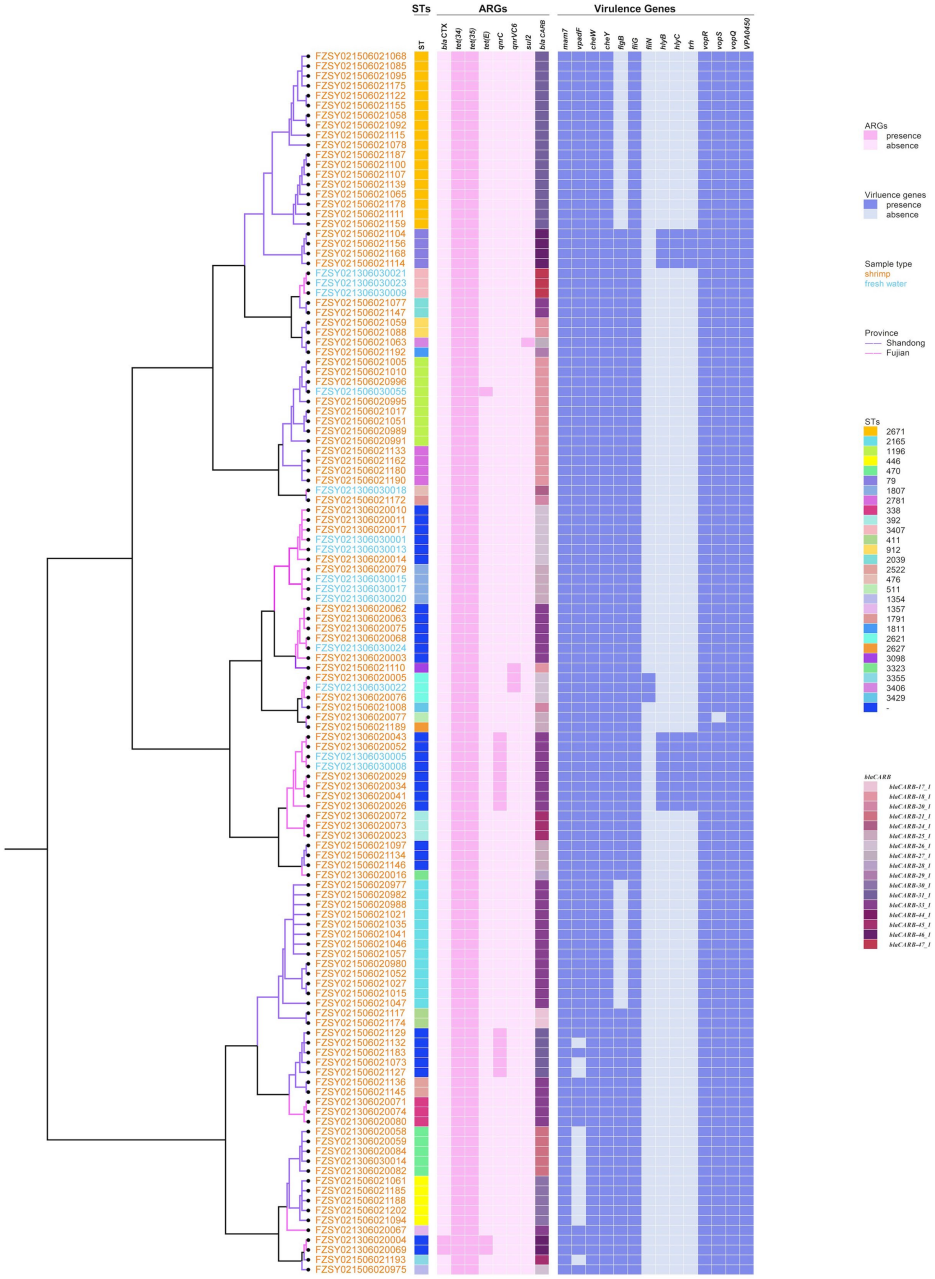
Phylogenetic tree and heatmap of sequence types, ARGs and virulence genes in 124 *V. parahaemolyticus* isolates. The different colors of branches and strain names represent the provincial origins and sample types of the strains, respectively. Different colors of squares were used to indicate the STs, ARGs and virulence genes.

Phylogenetic analysis demonstrated that ST significantly correlated with the 17 distinct *bla*_CARB_ gene variants, whereas isolates of the same ST harbored identical *bla*_CARB_ gene variants. Furthermore, this study identified four *V. parahaemolyticus* strains isolated from shrimps; the strains harbored the *trh* gene and all belonged to ST79, with the *bla*_CARB_ type being 46. Our study demonstrated that *hlyB* and *hlyC* genes encoding α-hemolysin were exclusively detected in *trh*+ strains and *trh*+ was coexisted with *hlyB* and *hlyC* (Figure 5).

## 4 Discussion

*V. parahaemolyticus* is an important foodborne pathogen, which is widely distributed in seawater and various seafood products, including shellfish and shrimp (Li et al., 2019). With increasing global demand for *L. vannamei*, freshwater aquaculture of this species has gained significant momentum in China. Study has demonstrated that *V. parahaemolyticus* is more prevalent in freshwater food than in seafood (Li et al., 2023). Therefore, the present study investigated the contamination characteristic of *V. parahaemolyticus* in freshwater-farmed *L. vannamei* from selected regions of China and characterized the genetic features of the isolates.

The results of the present study revealed a detection rate of 39.1% of *V. parahaemolyticus* in freshwater-farmed *L. vannamei* samples, which is considerably lower than the prevalence reported in most marine shrimp farming countries (Mok et al., 2021; Narayanan et al., 2020; Siddique et al., 2021; Tran et al., 2018), including India (80.0%), South Korea (54.5%), Bangladesh (69.4%), and Vietnam (87.5%), which rate was significantly higher than that observed in Zhejiang Province (3.33%) (Jiang et al., 2020), where shrimps are farmed in freshwater. However, the detection rate of *V. parahaemolyticus* in shrimp samples from Fujian Province (55.9%) was significantly higher than that in shrimp samples from Shandong Province (35.3%). This discrepancy may be attributed to the relatively higher salinity of aquaculture water in Fujian (3.65‰), compared to that in Shandong (0.53‰). *V. parahaemolyticus* abundance rises with increased salinity in freshwater, additionally, the abundance is associated with temperature and pH (León Robles et al., 2013). Furthermore, MLST based on seven housekeeping genes (González-Escalona et al., 2008) was performed on 94 *V. parahaemolyticus* isolates, identifying 28 STs. This demonstrates the genetic diversity of the strains from freshwater aquaculture sources. Notably, 24.3% of the strains (30/124) belonged to novel sequence types, suggesting potential genetic divergence of *V. parahaemolyticus* from freshwater aquaculture.

In the present, all *V. parahaemolyticus* isolates carried tetracycline resistance genes *tet(34)* and *tet(35)*, as well as β-lactam resistance gene *bla*_CARB_. Antibiotic resistance gene that *tet(34)* and *bla*_CARB_ are present in two *Vibrio parahaemolyticus* strains isolated from cultured shrimp in Bangladesh (Ahmmed et al., 2019). The detection of *bla*_CARB_ gene in this study is higher than that in isolates from farmed *L. vannamei* in Ningde (18.37%) (Zhang et al., 2024). Studies (Bai et al., 2024; Zheng et al., 2025) have demonstrated that the isolates from seafood harboring *tet(34)*, *tet(35)* and *bla*_CARB _46_ were susceptible to tetracycline but resistant to ampicillin and cefazolin. Furthermore, this study revealed a strong correlation between *bla*_CARB_ gene variants and STs, whereby isolates sharing identical STs carried the same *bla*_CARB_ variant. Consistent with previous reports (Li et al., 2016), *bla*_CARB_17_ demonstrated higher conservation than the commonly used *V. parahaemolyticus*-specific marker tlh in PCR detection, exhibiting 100% specificity. Based on these findings, we hypothesized that *bla*_CARB_ gene may serve as potential target for subtyping of *V. parahaemolyticus*, as an useful complementary method for MLST. Besides tetracycline and β-lactam resistance genes, there have quinolone and sulphonamide resistance genes absenced in aquaculture isolates, which may be its extensive use in human medicine and aquaculture systems (Lei et al., 2020). The application of biofertilizers increases fluoroquinolone resistance (Zhao et al., 2020).

The pathogenic factors of *V. parahaemolyticus* include hemolysins and T3SS and so on. Studies have shown that *tdh* and *trh*, which are closely associated with pathogenicity, are present in more than 80% of clinical isolates, whereas their carriage rates in food and environmental isolates generally range from 0 to 10% (León-Sicairos et al., 2022; Tewawong et al., 2024; Zhong et al., 2022). In this study, none of the strains carried the *tdh* gene, the *trh* positively rate among the shrimp-derived strains was 9.1% (10/110), which was lower than that in India saline water shrimps (9.5, 14.8%) (Sudan et al., 2023; Narayanan et al., 2020). Study (Paria et al., 2021) had revealed that the *tdh* and *trh* positive isolates were resistant to β-lactam antibiotics. Besides, our investigation revealed that the α-hemolysin genes *hlyB* and *hlyC* were exclusively present in *trh*-positive strains. This finding agrees with that of Zha et al. (2023), who identified a pathogenic *V. parahaemolyticus* strain lacking *tdh* and *trh* but carrying *hlyA*-*hlyD*. Combined with our results, we speculate that *hlyB* and *hlyC* represent unique hemolysins with potential pathogenic significance. Furthermore, all four *trh*+ strains belonged to ST79, which originated from shrimp samples collected from two distinct farms in Shandong Province. Because ST79 has also been reported in clinical isolates from China in PubMLST, it is important to confirm the potential risks of this ST through further investigation.

T3SS encompasses T3SS1 and T3SS2 clusters (Matsuda et al., 2020). Almost all the clinical and environmental strains of *V. parahaemolyticus* encode T3SS1, and T3SS2 is closely associated with its pathogenicity. In the present study all strains harbored T3SS1, which is consistent with the findings of Siriphap A (Siriphap et al., 2024). In contrast, none of the strains harbored the T3SS2 gene. Notably, one shrimp-derived strain from Fujian Province harbored genes encoding PirA and PirB toxins, which have been shown to cause acute hepatopancreatic necrosis disease (AHPND) in shrimp, with mortality rates of 70 ~ 100% (Lin et al., 2017), potentially leading to substantial economic losses in aquaculture. In total, our strains harbored 52 virulence-associated genes. The presence of these genes not only poses a potential public health threat but also threatens the sustainability of aquaculture.

In conclusion, the high detection rate of *V. parahaemolyticus* in Fujian freshwater-farmed *L. vannamei*. and the presence of virlence gene *trh* and β-lactam resistance genes in isolates have raised significant public health concerns. Besides, *V. parahaemolyticus* isolates in this study showed the emergence of ST79 co-harboring critical virulence genes *trh* and ARGs. Therefore, it is strongly recommended that targeted surveillance and specified control measures are being implemented in aquaculture systems in the future.

## Data Availability

The data presented in the study are deposited in the China National Center for Bioinformation repository, accession number is PRJCA051154.

## Footnotes

1
https://github.com/ablab/spades

2
https://github.com/Ecogenomics/CheckM

3
https://github.com/ParBLiSS/FastANI

4
https://quast.sourceforge.net/

5
https://cge.food.dtu.dk/services/KmerFinder/

6
https://pubmlst.org/organisms/vibrio-parahaemolyticus

7
https://github.com/achtman-lab/GrapeTree/releases

8
http://genepi.food.dtu.dk/resfinder

9
https://www.mgc.ac.cn/VFs

10
https://cge.food.dtu.dk/services/PlasmidFinder/

11
https://github.com/tseemann/snippy

12
https://github.com/nickjcroucher/gubbins

13
http://tree.bio.ed.ac.uk/software/figtree/
