# Correction: Prevalence, bacterial load, and genomic characterization of *Vibrio parahaemolyticus* in freshwater-farmed *Litopenaeus vannamei* from China

**DOI:** 10.3389/fmicb.2026.1766260

**Published:** 2026-01-26

**Authors:** Feifei Shan, Weiwei Li, Zunhua Chu, Peibin Hou, Na Sun, Ronghua Li, Xuejie Liu, Weiwei Chen, Ling Zhong, Shaofei Yan, Shenghui Cui, Yunchang Guo

**Affiliations:** 1 Institute for Nutrition and Health, Chinese Center for Disease Control and Prevention, Beijing, China; 2 Key Laboratory of Food Safety Risk Assessment, Food Safety Research Unit (2019RU014) of Chinese Academy of Medical Science, China National Center for Food Safety Risk Assessment, Beijing, China; 3 Shandong Center for Disease Control and Prevention, Jinan, China; 4 Jining Center for Disease Control and Prevention, Jining, China; 5 Fujian Center for Disease Control and Prevention, Fuzhou, China; 6 Zhangzhou Center for Disease Control and Prevention, Zhangzhou, China; 7 National Institutes for Food and Drug Control, Beijing, China

**Keywords:** *Vibrio parahaemolyticus*, *Litopenaeus vannamei*, prevalence, bacterial load, genomic analysis

There was a mistake in Figure 3 and Figure 5 as published. The figure 5 does not match the proof version, and figure 3 was uploaded incorrectly. The corrected figure 3 and figure 5 appears below.

**Figure 3:**
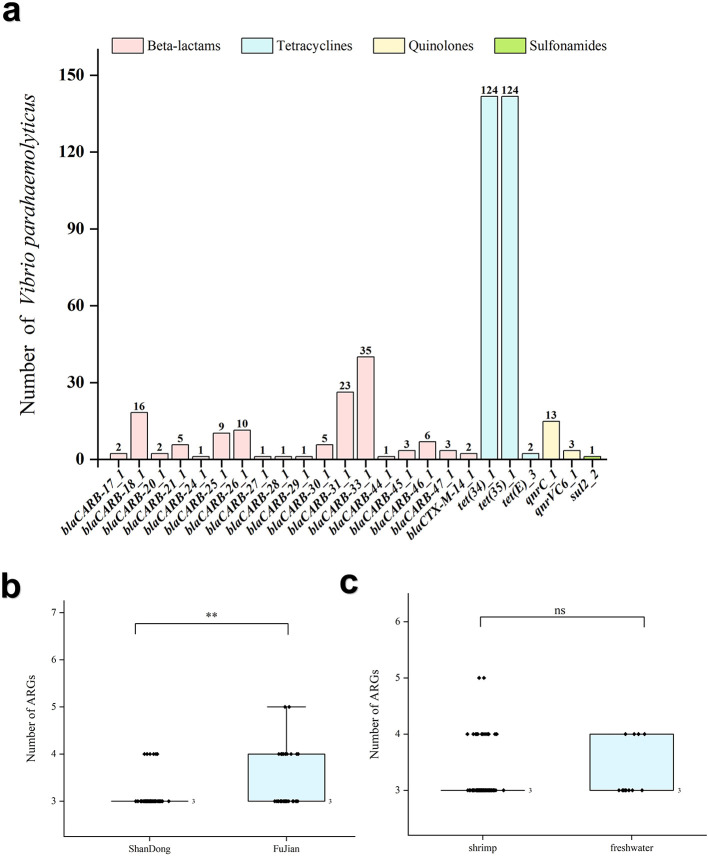
Distribution and statistical analysis of ARGs in 124 *V. parahaemolyticus* isolates. **(a)** Distribution of ARGs in 124 *V. parahaemolyticus* isolates. **(b)** Differences in the number of ARGs in isolates across different provinces. **(c)** Differences in isolates across number of ARGs in different sources. ‘^**^' means *P* < 0.01, ‘^***^' means *P* < 0.001, ‘ns' means *P* > 0.05.

**Figure 5:**
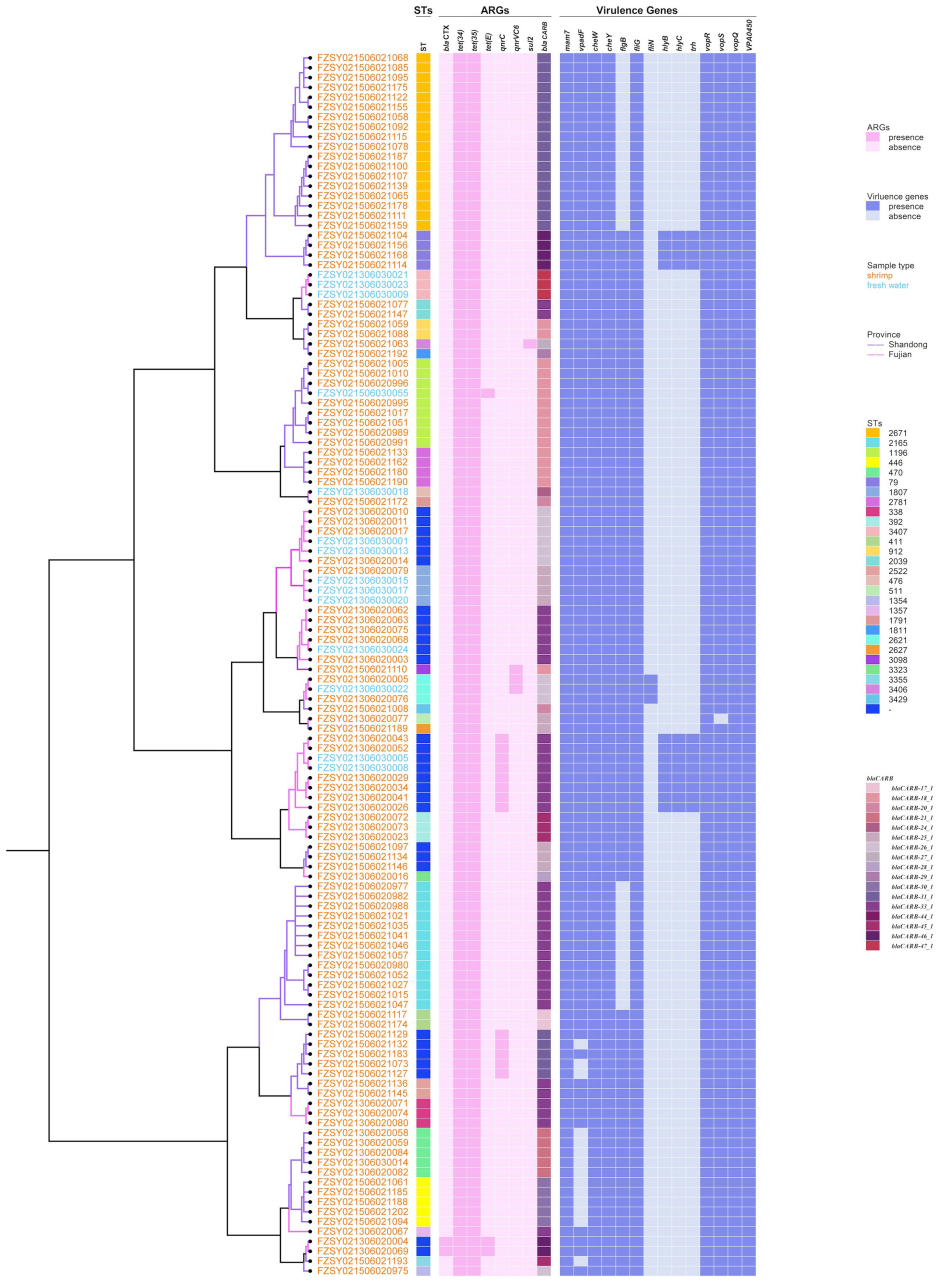
Phylogenetic tree and heatmap of sequence types, ARGs and virulence genes in 124 *V. parahaemolyticus* isolates. The different colors of branches and strain names represent the provincial origins and sample types of the strains, respectively. Different colors of squares were used to indicate the STs, ARGs and virulence genes.

There was a mistake in the caption of figure 2 as published. The caption of figure 2 does not match the proof version. The corrected caption of figure 2 appears below. “**Figure 2**. MLST and minimum spanning tree of 94 *V. parahaemolyticus* isolates.”

There was a mistake in the **Abstract** section as published. There was a typo error “This study aimed to investigate and evaluate the contamination of *V. parahaemolyticus* in freshwater-farmed marine shrimp (*L. annamei*) in China.”

The corrected sentences appears below.

“This study aimed to investigate and evaluate the contamination of *V. parahaemolyticus* in freshwater-farmed marine shrimp (*L. vannamei*) in China.”

The original version of this article has been updated.

